# Comparative genomics reveals contraction in olfactory receptor genes in bats

**DOI:** 10.1038/s41598-017-00132-9

**Published:** 2017-03-21

**Authors:** Georgia Tsagkogeorga, Steven Müller, Christophe Dessimoz, Stephen J. Rossiter

**Affiliations:** 1 School of Biological & Chemical Sciences, Queen Mary University of London, Mile End Road, London, E1 4NS UK; 2 University College London, Gower Street, London, WC1E 6BT UK; 3 University of Lausanne, Biophore, 1015 Lausanne, CH Switzerland; 4 Swiss Institute of Bioinformatics, Biophore, 1015 Lausanne, CH Switzerland

## Abstract

Gene loss and gain during genome evolution are thought to play important roles in adaptive phenotypic diversification. Among mammals, bats possess the smallest genomes and have evolved the unique abilities of powered flight and laryngeal echolocation. To investigate whether gene family evolution has contributed to the genome downsizing and phenotypic diversification in this group, we performed comparative evolutionary analyses of complete proteome data for eight bat species, including echolocating and non-echolocating forms, together with the proteomes of 12 other laurasiatherian mammals. Our analyses revealed extensive gene loss in the most recent ancestor of bats, and also of carnivores (both >1,000 genes), although this gene contraction did not appear to correlate with the reduction in genome size in bats. Comparisons of highly dynamic families suggested that expansion and contraction affected genes with similar functions (immunity, response to stimulus) in all laurasiatherian lineages. However, the magnitude and direction of these changes varied greatly among groups. In particular, our results showed contraction of the Olfactory Receptor (OR) gene repertoire in the last common ancestor of all bats, as well as that of the echolocating species studied. In contrast, non-echolocating fruit bats showed evidence of expansion in ORs, supporting a “trade-off” between sensory modalities.

## Introduction

Gene gain and loss are expected to be a major source of genomic variation, and a principal driver of phenotypic diversity^1, 2^. Increasing evidence from whole-genome sequencing has further corroborated this hypothesis, with multiple reported cases of gene family expansion underlying evolutionary innovations in animals^3^. Concurrently, large-scale population genomic studies in humans have shown that gene copy number variation can result in a range of pathologies or diseases^4^, highlighting further the contribution of changes in gene family size on phenotype^5^.

Mammalian genome evolution is characterized by multiple episodes of expansion and contraction^6^. A mammalian genome contains an average of around 3.14 Gb, however, genome size can reach 6.18 Gb in some members of the Afrotheria^7^. Of all mammals, bats possess the smallest genomes, with an average recorded content of 2.36 Gb ( ± 0.28 Gb S.E.), and a recorded minimum size of ~1.59 Gb (or 1.63 pg) in Carriker’s round-eared bat *Lophostoma carrikeri* ^8^. It was previously shown that genome size reduction in bats can be partially attributed to shortened introns and intergenic regions, a trend that is also seen in birds^9^. In both groups, it has been suggested that genome contraction might be an adaptation for powered flight and its associated high metabolic rates^10^.

In terms of the actual gene content, insights into the dynamics of gene gain and loss come mainly from genomic studies based on large-scale sequencing projects. Comparative analysis of the genomic sequences of the mouse-eared bat, *Myotis davidii*, and black flying fox, *Pteropus alecto*, with a range of other mammals, identified episodes of gene expansion in 71 gene families in *M*. *davidii* and 13 in *P*. *alecto*, as opposed to 41 and 35 contractions, respectively^11^. Likewise, it has been reported that 44 families have experienced gene loss, and 67 gene expansion, in the genome of the Brandt’s bat *Myotis brandtii* ^12^.

Despite the large amount of genomic data generated for bats over the past four years^11–13^, a comprehensive picture of gene gain and loss at a genome scale for the group is still lacking. This is mainly because all studies so far have focussed on one or two representative species at a time, or, when encompassing more taxa, have been restricted to specific gene families^14–16^. Here we investigate the patterns of gene family evolution in bats in greater depth. Using a comparative genomics approach spanning 20 mammalian genomes, we assess the average turnover of gene gain and loss in bats, and test whether this rate is different from that of other closely related lineages. We also aim to identify families that have undergone accelerated evolution in the last common ancestor of bats, as well as in the last common ancestor of echolocating and non-echolocating forms. Finally, we address the question of whether rates of change in gene family size in bats are associated with their unusually small genome sizes.

## Results and Discussion

### Taxon sampling and inference of Hierarchical Orthologous Groups (HOGs) across mammals

To identify homologous genes and elucidate evolutionary patterns of gene gain and loss in bats, we compared the complete proteomes from 20 laurasiatherian mammals using the Orthologous MAtrix (OMA) algorithm implemented in the OMA standalone software package^17^. Our sampling included eight bats, of which three are non-echolocating Old-World fruit bats from the suborder Yinpterochiroptera (*Pteropus alecto*, *P*. *vampyrus* and *Rousettus aegyptiacus*), and five are echolocating species from the suborder Yangochiroptera (*Miniopterus natalensis*, *Eptesicus fuscus*, *Myotis brandtii*, *M*. *davidii* and *M*. *lucifugus*). To assess completeness of the laurasiatherian proteomes, we used the software BUSCO^18^. The estimated completeness across our sampled species gene annotations varied from 55% for *Sorex araneus* to 98% for *P*. *alecto*. All bat proteomes were 90% complete, with levels of fragmentation varying from 1% for *P*. *alecto* and *R*. *aegyptiacus* to 4.9% for *M*. *davidii* (Supplementary Table S1).

**Figure 1:**
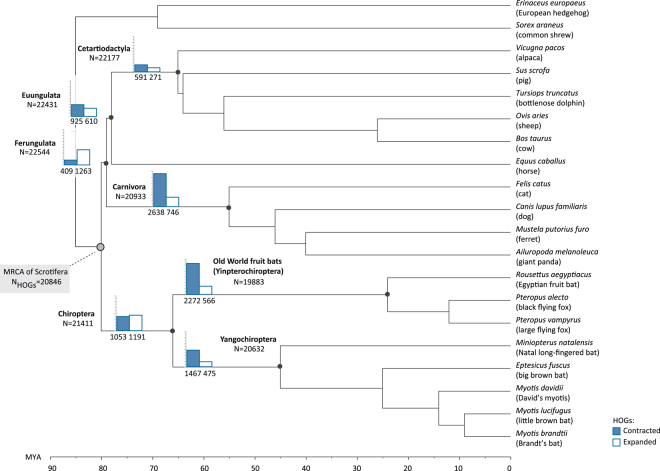
Gene losses and gains along key branches of the Laurasiatheria phylogeny, as inferred from OMA hierarchical groups.

For our OMA analysis, we used the species tree topology based on the most recent phylogenomic study of bats^13^, in which bats were found to be most closely related to the Ferungulata, i.e., the clade uniting carnivores and ungulates (Fig. 1). However, the phylogenetic relationship among laurasiatherian lineages remains highly contentious, attributed largely to their rapid radiation ~80 million years ago and associated extensive incomplete lineage sorting^13^. As a result, many competing phylogenetic scenarios have been proposed for the diversification of laurasiatherian mammals, and thereby the phylogenetic positioning of bats within them^19–25^. To account for the effects of species phylogeny on inferring gene gains and losses in bat genomes, we also repeated our analyses under six proposed alternative species tree topologies for Laurasiatheria (Supplementary Figure S1), drawn from the recent literature^19–25^.

Using all-against-all similarity searches coupled with a graph-based clustering approach, OMA grouped the 20 mammalian proteomes into 20,936 Hierarchical Orthologous Groups (HOGs—sets of all genes descended from a common ancestral gene within the Laurasiatheria). We inferred 32,571 orthologous genes across all sampled species, including 26,537 with at least one bat sequence.

### Gene gain and loss in bats and other major laurasiatherian lineages

Based on HOG information, we mapped gene duplications and losses on our mammalian tree (Fig. 1). Considering key branches of our phylogeny, our analyses identified 14,445 HOGs present in most recent common ancestor (MRCA) of all sampled mammals, and 20,846 HOGs in the branch leading to the MRCA of the clade uniting bats, carnivores and ungulates (clade of Scrotifera). We obtained 22,544 HOGs in the MRCA of carnivores and ungulates (clade of Ferungulata) and 21,411 HOGs in the MRCA of bats (Chiroptera) (Fig. 1).

Inference of ancestral gene duplication events across Laurasiatheria revealed 1,191 episodes of ancestral gene expansion in the MRCA of bats, resulting in 2,678 multi-copy genes since their divergence from carnivores and ungulates (MRCA of Scrotifera in Fig. 1). Very similar levels of gene duplication were also inferred for the latter two groups, with 1,263 expansions predicted at the level of the MRCA of Ferungulata (Fig. 1; Supplementary Table S2).

In terms of gene loss, the MCRA of bats showed evidence of having undergone more extensive loss compared to that of the Ferungulata, with 1,191 gene losses compared to 409, respectively. Within the Ferungulata, high numbers of HOG contractions were also inferred along the branch leading to the MRCA of carnivores (n = 2,638). The fewest changes in gene family size were observed along the branch of the MRCA of Cetartiodactyla, with 591 HOGs showing evidence of gene loss, nearly half of that predicted in bats (Fig. 1; Supplementary Table S2).

### Functional profiling of expanded and contracted gene families in bats

To examine the biological role of HOGs that show changes in number in bats, we first assigned one representative member of each HOG to GO terms in UniProtKB^26^ (Supplementary Tables S3–S5). Among HOGs that appeared to have expanded, the vast majority were categorised as being genes involved in biological regulation (>400 HOGs), metabolic genes (>300 HOGs), and genes associated with a response to stimulus (>200 HOGs). We also obtained a strong signal of expansion for genes involved in developmental process (>100 HOGs), cellular component organization (>100 HOGs), immunity (80 HOGs), locomotion (>40 HOGs), and reproduction (>40 HOGs) (Supplementary Table S4). Similar functional profiles were obtained for HOGs showing evidence of gene loss in bats, with again a substantial number of gene clusters linked to metabolic process (>300 HOGs) and response to stimulus (~200 HOGs), as well as cellular localisation (>100 HOGs), developmental process (>100 HOGs) and immune system (>50 HOGs) (Supplementary Table S4).

**Figure 2:**
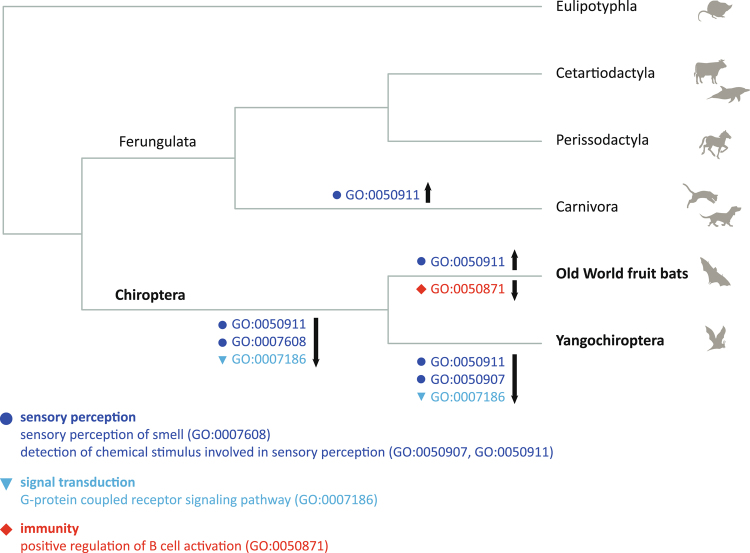
Significant GO terms associated with expanded and contracted HOGs in bats and other laurasiatherians (Ontology: Biological Process; Bonferroni corrected p-value < 0.05). Arrows indicate expansion (upward) and contraction (downward).

To gain further insights into putative roles of genes duplicated and lost in bat genomes, we looked at the annotation of their homologues in cow and human genomes (Supplementary Table S3) and carried out GO enrichment analyses to test for overrepresented functional terms associated with expanded and contracted HOGs in bats (Supplementary Tables S6 and S7). We did not find any significantly enriched GO terms for expanded genes in the MRCA of bats after correction for multiple testing (Supplementary Table S6), however, our analyses indicated that genes encoding proteins with olfactory receptor activity (GO:0004984), including those involved in the sensory perception of smell (GO:0050911), were significantly enriched among contracted HOGs in bat genomes (Fig. 2, Supplementary Table S7). This reduction in numbers of OR genes was corroborated using both the cow (uncorrected p-value = 3.26E-10; p-value Bonferroni corrected = 4.34E-06) and human homologues (uncorrected p-value = 8.70E-11; p-value Bonferroni corrected = 1.42E-06). GO results based on human also showed significant enrichment (p-value Bonferroni corrected <1E-05) in genes associated with G-protein coupled receptor activity (GO:0004930) and signaling pathway (GO:0007186), sensory perception of smell (GO:0007608), as well as in genes with products categorised as odorant binding (GO:0005549 in Supplementary Table S7).

Of all 1,053 HOGs inferred as being reduced in size in bats, we identified 357 HOGs that appeared to be completely absent in bats (i.e. with no homologous sequences detected). Of these, a few consisted of ambiguous groupings comprising two to three (usually partial) sequences from other mammal genomes that presumably failed to cluster together with the remaining HOGs. To filter genuine signal of gene loss from potential clustering artifacts, we identified HOGs that contained no bat genes yet included a sequence from at least three of the laurasiatherian lineages sampled (i.e. from Carnivora, Cetartiodactyla, Perissodactyla and Eulipotyphla). We identified 201 such cases in total, with most having functional links with biological regulation (92 HOGs), response to stimulus (83 HOGs), and signal transduction (76 HOGs). We also performed clustering of these HOGs using a keyword clustering approach from protein description available in UniProtKB, which recovered associations for 73 HOGs to biological processes of olfaction (68), transcriptional regulation (3), differentiation (1), myogenesis (1) transport (1) and Ubl conjugation pathway (1). Looking at the protein identifiers *per se*, we confirmed that the vast majority of HOGs with no orthologues in bats consisted of Olfactory Receptor (OR) genes (Supplementary Table S5).

For any given lineage, identifying genes that are novel is inherently more difficult than identifying ones that are either lost or duplicated. This is because novel genes are usually not associated with any functional annotation, and also because they might be present in unsampled taxa. Thus to characterise putative novel genes present only in bats, we manually inspected representative gene identifiers for corresponding HOGs and functionally classified these loci either on the basis of similarity to other known genes (e.g. “MHC class II transactivator-like” gene) or the presence of common protein motifs (e.g. zinc finger protein like). We identified 131 HOGs containing putatively novel genes, 37 corresponded to low quality or/and uncharacterised proteins. Of the remaining HOGs, at least 11 had a binding activity, six corresponded to transport proteins, five had functional links to immunity, and two to sensory perception of olfaction. We further identified HOGs with putative novel genes encoding common protein domains, such as zinc finger (6), growth factors (2) and IQ protein domains (3).

### Functional annotation of HOG size changes in other lineages

We next assessed whether the patterns of HOG expansion and contraction in bats with respect to gene types were also seen in other laurasiatherian groups. For this, we conducted GO enrichment analyses of HOGs showing expansion and contraction in the MRCAs of Ferungulata, and its constituent lineages Cetartiodactyla and Carnivora (Supplementary Tables S8–S13). We found no significant GO terms after correcting for multiple tests in the MRCAs of Ferungulata and cetartiodactyl mammals. In contrast, however, genes encoding proteins with olfactory receptor activity (GO:0004984) associated with the sensory perception of smell (GO:0050911) were enriched among HOGs showing expansion in the MRCA of carnivores (uncorrected p-value = 8.38E-09; p-value Bonferroni corrected = 0.0001); this trend in carnivores was thus opposite to that observed in bats (Fig. 2, Supplementary Table S10). In the same branch, our results suggested expansion also in genes encoding for ribosomal proteins (uncorrected p-value = 1.50E-06; p-value Bonferroni corrected = 0.0244). Some evidence for expansion in OR genes was also detected for the MRCAs of Ferungulata and Cetartiodactyla although with low statistical support (uncorrected p-value = 0.0004 in Supplementary Table S8, and uncorrected p-value = 0.0080 in Supplementary Table S12). We found no evidence of enrichment associated with sensory perception genes for contracted HOGs along the ancestral branches of Ferungulata, Carnivora or Cetartiodactyla (Supplementary Tables S9, S11 and S13).

### HOG evolution in echolocating and non-echolocating bats

Within bats, our analyses inferred 19,883 HOGs in the MRCA of the three non-echolocating Old-World fruit bats, *R*. *aegyptiacus*, *P*. *alecto* and *P*. *vampyrus*, and 20,632 in the MRCA of the clade uniting the echolocating bats *M*. *natalensis*, *E*. *fuscus*, *M*. *brandtii*, *M*. *davidii* and *M*. *lucifugus*, all of which are members of the suborder Yangochiroptera (Fig. 1).

In terms of gene expansion, we inferred 1,283 and 1,076 multi-copy genes in the ancestral branches of Old World fruit bats and Yangochiroptera, respectively, which were clustered into 566 and 475 respective HOGs (Fig. 1; Supplementary Table S2). Functional annotation of expanded HOGs using UniProtKB suggested an increase in genes involved in biological regulation, metabolic processes, response to stimulus, development, localization, and immunity in both echolocating and non-echolocating bat genomes (Supplementary Table S14).

GO enrichment analyses based on human homologues showed a significant expansion in OR genes (GO:0050911, GO:0004984) in the MRCA of Old World fruit bats (uncorrected p-value = 3.99E-11; p-value Bonferroni corrected = 6.50E-07), which was not found in the MRCA of the echolocating species examined (Fig. 2, Supplementary Tables S15 and S16). Similar results, were also observed when annotation was based on cow homologues, together with expansion in genes involved in respiratory system process (GO:0003016) and the regulation of defense response to viruses (GO:0050691), although all four terms showed low statistical support after correction (Supplementary Table S15). Top GO terms for HOGs expanded in Yangochiroptera genomes suggested expansion in genes linked to cell fate determination (GO:0007493, GO:0042074), lipid digestion (GO:0044241) and the regulation of immune response (GO:0050776), although again with low statistical support (Supplementary Table S16).

Compared to gene gain (n = 566), we detected a four-fold increase in gene loss in Old World fruit bats (n = 2,272) (Fig. 1; Supplementary Table S2). HOGs showing contraction along the MRCA of Old World fruit bats were associated with 50 GO terms based on human homologues (uncorrected p-value < 0.01, Supplementary Table S15). Of these, top ranked gene clusters had a direct link with immunity and pathogen recognition (GO:0050871, GO:0003823, GO:0006958, GO:0034987, GO:0042571, GO:0006910, GO:0059776,). In particular, our analyses suggested a significant enrichment of genes involved in B cell activation (GO:0050871, uncorrected p-value = 1.00E-06; p-value Bonferroni corrected = 0.0163) and antigen binding (GO:0003823, uncorrected p-value = 2.88E-06; p-value Bonferroni corrected = 0.0468) among HOGs showing contraction along the branch of the MRCA of Old World fruit bats (Fig. 2, Supplementary Table S17). Other contracted HOGs in this group were associated with metabolism (e.g. GO:0006706, GO:2001303). Functional annotation of contacted HOGs based on cow also suggested a potential contraction in genes involved in sensory perception of smell (GO:0007608), as well as genes associated with development and morphogenesis (e.g. GO:0061326, GO:0072144, GO:0003278, see Supplementary Table S18).

Echolocating bats from the suborder Yangochiroptera also showed greater gene loss (n = 1,467) than gain, although this was less pronounced than in the fruit bats (Fig. 1; Supplementary Table S2). GO analyses of gene losses along the ancestral branch of these bats firmly supported enrichment in genes involved in detection of chemical stimulus and sensory perception of smell (GO:0050911, p-value Bonferroni corrected = 9.57E-07), and for OR genes (GO:0004984, p-value Bonferroni corrected = 9.57E-07) in particular (Fig. 2, Supplementary Table S18). Robust statistical support was also obtained for contraction in the G-protein coupled receptor signaling pathway (GO:0004930, GO:0007186, p-value Bonferroni corrected <1E-08 in Supplementary Table S18).

Overall, while Old World fruit bats showed a much greater extent of gene contraction than did echolocating species, we cannot rule out the possibility that this difference might reflect differences in the sampling size of the two groups in our analyses (three Old World fruit bats sampled versus five yangochiropterans). Despite this, the functional profiles of genes lost in these two groups are distinctive from each other, and it is less plausible that these are driven solely by potential biases in the data (Supplementary Tables S17 and S18).

With regards to novel gene gain, we identified 27 putatively novel genes in the MRCA of Old World fruit bats. Among these, eight seem to encode for binding proteins and four are associated with a response to stimulus (two involved in defence and two in olfaction) and three with metabolism. In the MRCA of Yangochiroptera, our analyses inferred 87 putative gene gains, including functions related to immune system (~30), metabolism (~40) and responses to stimulus (~50).

### Effects of species phylogeny on gene expansions and contractions in bats

Our results showed that estimates of HOG expansions and contractions along the MRCA of bats varied widely among different tree topologies, with the number of gene expansions ranging from 350 to 1214 HOGs and gene losses from 768 to 1861 depending on the topology used (Supplementary Table S19). On the contrary, estimates of gene gain and loss in the MRCA of Old World fruit bats and that Yangochiroptera were relatively unaffected by the species tree, showing almost no variation across the six topologies tested (Supplementary Figure S1, Supplementary Table S19).

To assess further the robustness of our results across alternative topologies, we repeated the GO analyses for the two trees that showed the greatest differences in estimates of gene gain and loss, both compared to our results, and to each other (Trees #2 and #3 in Supplementary Figure S1 and Supplementary Table S19). These results also firmly supported a contraction in OR genes (GO:0004984, p-value Bonferroni corrected <2.3E-06) and in genes involved in sensory perception of smell (GO:0050907, GO:0050911, p-value Bonferroni corrected <3.5E-05) in both the MRCA of bats and that of echolocating taxa (Supplementary Table S20). Moreover functional annotation of HOGs corroborated both the observed expansion of olfactory genes in Old World fruit bats and Carnivores (GO:0004984, GO:0050911, corrected p-values < 4.8E-04) (Supplementary Table S21). Finally, under the two alternative trees we found a stronger signal for changes in immunity genes for across all lineages tested (Supplementary Tables S20 and S21), in line with expectations that immunity-related gene families show dynamic patterns of evolution in mammals.

### Maximum likelihood estimation of genomic turnover

Although the mechanism by which multiple gene copies are generated and maintained is debated, it has been shown that many gene families follow a birth-and-death mode of evolution, including immune gene families and sensory receptor superfamilies^27, 28^ According to this model, genes are created by gene duplication and some are maintained in the genome for a long time, whereas others are deleted or become nonfunctional through deleterious mutations^29^. In addition to this, draft genome assemblies are prone to extensive errors in predictions of number of genes due to genome fragmentation and thus poor quality gene annotations^30^, which may result in either an overestimation or underestimation of the gene load. An overestimation in the number of genes present in such genomes may be caused by the splitting of alleles into separate scaffolds or contigs. Conversely, the opposite may also happen whereby copy number variants or recent paralogues are erroneously collapsed together into a single genomic region, leading to an underestimation of gene copy number.

To account for potential erroneous estimates of the numbers of inferred homologous groups arising from the types of errors described above, we fitted to our data a recently developed model of gene family evolution that allows for estimation and correction of annotation errors from incomplete genomes^31^. We calculated the global error in our HOG dataset, as well as error for each species separately. Global error estimation in the 20,936 HOGs was found to be 0.0635, suggesting an average of 6.35% error in our HOG size measures at the tips of our tree. However, error estimates varied widely among sampled genomes, with individual species error ranging from 0 (e.g. *M*. *natalensis*) to 0.4126 (e.g. *Vicugna pacos*). Aside from the *M*. *natalensis* data, error for the remaning bats spanned from 0.0130 for *E*. *fuscus and R*. *aegyctiacus* to 0.1396 for the *M*. *lucifugus* genome.

To assess the average rate of gene gain (λ) and loss (μ) at a genome-scale and across our entire tree, the bat clade, as well as in the Old World fruit bats and Yangochiroptera, we analysed a subset of 18,698 HOGs that had at least one representative member present in the MRCA of Scrotifera (bats + carnivores + perissodactyls + cetartiodactyls) in a ML framework. Using birth-and-death models, we first estimated the expected number of changes in HOG size across our tree. Assuming equal rates of gene gain and loss, and accounting for errors at an individual species level, the average rate of gene turnover across our laurasiatherian phylogeny was estimated to be 0.0008 changes/gene/million years (Mya) (Table 1, one-lambdamu model + individual species error correction). Breaking this down further, we obtained estimates of the global rates of gene duplication (λ = 0.0001 duplications/gene/Mya) and loss (μ = 0.0016 losses/gene/Mya) (see Table 1, one-lambdamu model + individual species error correction).

**Table 1:** Overview of birth-and-death (BD) models fitted on 18,698 HOGs present in the MRCA of Scrotifera (Bats + Carnivores + Perissodactyla + Cetartiodactyla) estimated using the OMA pipeline.

BD Model	λ_0_	λ_1_	λ_2_	μ_0_	μ_1_	μ_2_	-lnL	#HOGs p* < 0.05	#HOGs p* < 0.01	#HOGs p* < 5.35e-7
**No error correction**										
1-lambda	λ_0_ = 0.0017	—	—	—	—	—	184,044.80	3,032	1,937	188
2-lambda	λ_0_ = 0.0017	λ_bats_ = 0.0018	—	—	—	—	184,028.60	3,129	1,923	518
3-lambda	λ_0_ = 0.0016	λ_yan_ = 0.0021	λ_owf_ = 0.0017	—	—	—	183,868.78	2,904	1,781	1,010
1-lamdamu	λ_0_ = 0.0004	—	—	μ_0_ = 0.0030	—	—	167,963.90	3,357	1,945	851
2-lamdamu	λ_0_ = 0.0003	λ_bats_ = 0.0004	—	μ_0_ = 0.0029	μ_bara_ = 0.0031	—	167,925.19	3,352	2,368	875
3-lamdamu	λ_0_ = 0.0008	λ_yan_ = 0.0007	λ_owf_ = 0.0005	μ_0_ = 0.0029	μ_yan_ = 0.0035	μ_owf_ = 0.0030	167,644.44	3,444	2,118	614
**Global error correction**, **ε** = **0**.**0635**										
1-lambda	λ_0_ = 0.0008	—	—	—	—	—	170,416.03	3,126	2,200	1,285
2-lambda	λ_0_ = 0.0010	λ_bats_ = 0.0006	—	—	—	—	170,086.71	4,673	3,592	2,817
3-lambda	λ_0_ = 0.0011	λ_yan_ = 0.0004	λ_yan_ = 0.0007	—	—	—	169,283.74	4,995	3,924	2,834
1-lamdamu	λ_0_ = 0.0001	—	—	μ_0_ = 0.0019	—	—	160,043.58	5,346	4,182	2,421
2-lamdamu	λ_0_ = 0.0001	λ_bats_ = 0.0001	—	μ_0_ = 0.0021	μ_bara_ = 0.0014	—	159,679.42	5,304	3,920	2,732
3-lamdamu	λ_0_ = 0.0001	λ_yan_ = 0.0001	λ_owf_ = 2.24e-05	μ_0_ = 0.0023	μ_yan_ = 0.0010	μ_owf_ = 0.0008	159,082.23	5,597	4,429	2,799
**Individual species error correction**, **ε** = **[0**, **0**.**4126]**										
1-lambda	λ_0_ = 0.0008	—	—	—	—	—	162,987.48	5,076	3,980	2,336
2-lambda	λ_0_ = 0.0008	λ_bats_ = 0.0008	—	—	—	—	162,987.36	5,144	4,259	2,921
3-lambda	λ_0_ = 0.0008	λ_yan_ = 0.0007	λ_owf_ = 0.0005	—	—	—	162,836.70	5,006	3,682	2,746
1-lamdamu	λ_0_ = 0.0001	—	—	μ_0_ = 0.0016	—	—	155,133.69	5,431	3,806	1,503
2-lamdamu	λ_0_ = 0.0001	λ_bats_ = 0.0001	—	μ_0_ = 0.0017	μ_bara_ = 0.0015	—	155,107.15	5,192	4,093	2,699
3-lamdamu	λ_0_ = 0.0001	λ_yan_ = 0.0002	λ_owf_ = 0.0001	μ_0_ = 0.0018	μ_yan_ = 0.0013	μ_owf_ = 0.0011	154,901.51	5,499	4,517	2,547

*Describes the likelihood of the observed sizes given the rates of gain and loss.

We confirmed that the estimate of gene turnover was much higher when error in the data was not taken into consideration in the ML analyses (one-lambda model with no correction: λ = μ = 0.0017) or when only global error was accommodated (one-lambda model + global error correction: λ = μ = 0.0008). The model that incorporated error at a species-level gave the best fit to our data based on the Akaike Information Criterion (AIC) (Supplementary Table S22).

We also sought to estimate the average rate of gene turnover for the bat clade using two-lambda models. Our results suggested an equal average genomic turnover for bats compared to the rest of the Laurasiatheria after accommodating potential error (λ_bats_ = μ_bats_ = 0.0008). Although the observed turnover for bats and the rest of laurasiatherian mammals was estimated to have the same value, a model comparison based on simulations (Supplementary Figure S2) and the AIC supported a higher probability of a gene being lost in the other laurasiatherians than in bats (best model two-lambdamu: λ_bats_ = 0.0001, λ_0_ = 0.0001; μ_bats_ = 0.0015, μ_0_ = 0.0017; ΔLnl = 26.54 [critical value 3.627, p = 0.05] in Supplementary Figure S2, lowest AIC in Supplementary Table S22). A similarly lower rate of gene loss was also detected in our control analyses for carnivores (λ_carnivores_ = 0.0001, λ_0_ = 0.0001; μ_carnivores_ = 0.0014, μ_0_ = 0.0017), whereas cetartiodactyls showed the inverse relationship, *i*.*e*., an increased rate of average gene loss (two-lambdamu: λ_cetartiodactyls_ = 0.0001, λ_0_ = 0.0001; μ_cetartiodactyls_ = 0.0019, μ_0_ = 0.0016, in Supplementary Table S22).

Finally, we looked at the rates of change in HOG size within bats by fitting a three-lambda model, in which we specified different rates of gene family turnover for non-echolocating Old World fruit bats (λ_owf_), for echolocating forms from the suborder Yangochiroptera (λ_yan_), and for the rest of the tree (λ_0_). With and without error correction, our results suggested a large difference in the amount of gene gain and loss between echolocating and non-echolocating forms, with genomic turnover being higher in the former (suborder Yangochiroptera λ_yan_ = 0.0007) compared to the latter (Old World fruit bats λ_owf_ = 0.0005). This trend held when rates of gene expansion were separately estimated from rates of contraction (best-fit model: 3-lamdamu + species error correction in Table 1), with estimates of both λ_yan_ and μ_yan_ being higher that corresponding values of λ_owf_ and μ_owf_.

To assess whether the observed gene turnover in bats could help to explain their small genome sizes, we calculated the average gene expansion and expected number of gene gains and losses for each of the eighteen terminal branches of our phylogeny under the one-lambda model with error correction at a species-level. Correlating these estimates with respective genome sizes revealed no significant correlation, either before or after accounting for phylogenetic affiliation. Thus we found no evidence that broad trends in gene loss and gain have contributed to genome contraction in bats, at least for the set of taxa and the 18,698 groups of homologues studied.

### Accelerated evolution of HOGs in echolocating and non-echolocating bats

We calculated the probability for each HOG evolving under the stochastic birth-and-death process, the λ rate, as well as the mean number of gene gain or loss per HOG along each branch of the species tree (data not shown). Among 18,698 HOGs examined, we found that 2,336 were highly unlikely to have evolved under a random gain and loss process (corrected p-value < 0.01), instead showing evidence of accelerated evolution. Of these non-randomly evolving HOGs, we identified 533 showing rapid evolution along at least one bat branch (branch-specific p-value < 0.01), 130 in the Old World fruit bat clade and 460 in the clade of Yangochiroptera. Functional annotation of these 533 HOGs revealed putative associations with signal transduction (108), in particular in G-protein coupled receptors (75) such as OR and taste receptors. Evidence of accelerated evolution was also found in genes encoding proteins of the immune system (22), including endogenous retroviral elements, interferon, MHC, T-cell receptor families, and gene families involved in organ development (21) and reproduction (10).

For comparison, we examined non-randomly evolving HOGs in carnivores and cetartiodactyl mammals. We identified 248 HOGs showing accelerated evolution in the Carnivora and 537 in the Cetartiodactyla (branch-specific p-value < 0.01). Functional annotation of these groups revealed mainly metabolic and OR types of genes in carnivores, in line with our results of HOG expansion obtained from the OMA pipeline. Similar to bats, accelerated rates of gene turnover were also detected in immunity related genes and reproductive proteins in both Carnivora and Cetartiodactyla.

### Gene turnover and insights into bat biology

Our analyses of HOGs found that the most highly dynamic gene families in terms of turnover across all laurasiatherian lineages were related to olfaction. In particular, we found a strong signature of contraction of OR genes in all bats (Fig. 2, Supplementary Table S7), which was also associated with an accelerated rate of gene family evolution compared to a random birth-and-death process. Comparing echolocating and non-echolocating species, we found clear contraction of OR genes in the former and significant support for expansion in the latter (Fig. 2, Supplementary Tables S15–S18). OR genes typically form the largest gene family in mammalian genomes and show high variation among species in terms of number, as well as in their degree of pseudogenization^32, 33^. In New World fruit bats (family Phyllostomidae), variation in OR number appears to correlate with niche specialization^14^, while contraction in the OR gene repertoire in the *M*. *brandtii* genome was suggested to reflect an evolutionary shift from olfaction to echolocation^12^. Our findings of significant contraction in the root of all bats as well as in echolocating forms, lend some support to this idea, perhaps pointing to a “trade-off” between sensory modalities, although wider sampling, including echolocating forms from the Yinpterochiroptera, is needed to confirm this trend (see also refs 16, 34) (Supplementary Tables S15 and S16).

Surveys of HOGs across 20 mammalian genomes also revealed expansion in up to 80 orthologous genes with links to immunity in bats (although this high turnover might arise from the difficulties of annotating highly divergent loci). Bats are well-known as reservoirs for a range of zoonotic diseases^35^ and, as such, there have been a number of recent studies aimed at understanding the evolutionary dynamics of bat immunity genes^9, 12, 36, 37^. Previous work has reported molecular adaptation in DNA repair loci and innate immune pathways in bat lineages^11, 15^. Some immunity genes appear to have undergone expansion in bat genomes, including the leukocyte receptor complex (LRC) superfamily in *M*. *davidii* ^11^ and the *FBXO31* gene involved in ubiquitin-mediated degradation in *M*. *brandtii* ^12^. In our study, specific HOGs showing high levels of gene gain in bats included several transmembrane receptors implicated in immune responses, such as *CD-*, *CEA-* and *CR-*like proteins, glycoproteins (e.g. *AZGP1*) and proteoglycans (e.g. *PRG*-like genes). Interestingly, previous studies of bats have also revealed several cases of contraction in immunity genes; these include killer-cell immunoglobulin like receptors (KIRs), killer cell lectin-like receptors (KLRs or Ly49 receptors)^11^, IFN-α genes^37^, and PYHIN genes^11, 38^. Our analyses of HOGs revealed that the contraction of PYHIN, previously reported for *P*. *alecto* and *M*. *davidii*, is common to all eight bat species studied.

Across all five focal lineages tested in our study, the only significant GO enrichment for loci showing gene gain or loss was seen in the ancestral branch of Old World fruit bats, and involved the loss of immunity genes implicated in B cell activation and antigen binding (Fig. 2, Supplementary Table S17). However, looking at a wider taxonomic scale, it is noteworthy that the high inferred plasticity in numbers of immunity genes in bats was also seen in other mammalian lineages (Supplementary Tables S6, S7 and S8–S13, respectively). Some of these changes appeared to be associated with an accelerated rate of evolution, as revealed for immunoglobulin proteins based on our ML analyses of birth-and-death. Additional evidence is needed to establish whether differences in the gene complement are associated with variation in immune response to pathogens.

## Conclusions

We show that bat genomes are highly plastic with respect to the turnover of protein-coding genes, but that the rate of gene turnover appears to be similar to that of their close relatives within the Laurasiatheria. A strong trend of gene loss in ORs in bats, and echolocating lineages in particular, was the opposite to the trend seen in both the non-echolocating bats and the carnivores examined, suggestive of a potential trade-off between olfaction and other senses in auditory specialists. These findings appeared to be robust to a range of proposed tree topologies for the relationships among laurasiatherian lineages.

Overall, our findings indicate that gene turnover tends to involve families with similar functional profiles, notably loci involved in immunity, regulation, metabolism and responses to stimulus. One possible explanation for this could be that the current status quo of GO annotation is insufficient or over-generic to allow rigorous tests of associations between gene family changes and biological functions. It could also be that only a small number of genes are essential for phenotypic adaptation, and that the signature of changes in these within the genome is masked by changes in larger highly dynamic families, such as OR and immunity related genes. Alternatively, if the evolution of novel phenotypes is indeed mediated via gene duplication and/or loss, then the underlying mechanism may not necessary pertain to specific gene targets but may operate through the same highly plastic groups of genes.

## Methods

### Inference of homologues across bat and mammal genomes

To investigate the evolutionary dynamics of gene gain and loss in bats, we first identifed homologous genes among bats and other closely related laurasiatherian mammals using the OMA standalone software package^17^. The OMA pipeline clusters homologous sequences from complete genomes in order to identify orthologous pairs of sequences and infer hierarchical orthologous groups (“HOGs”) of genes that have descended for a common ancestral gene in a specified taxonomic range. OMA has been shown to be among the most reliable orthology inference methods—notably outperforming many tree-based methods (e.g. refs 39–41).

We augmented the two bat species contained in the May 2016 OMA database (*M.* lucifugus and *P.* vampyrus) with six bat proteomes retrieved from the GenBank (*E*. *fuscus*, *M*. *natalensis*, *M*. *brandtii*, *M*. *davidii*, *P*. *alecto* and *R*. *aegyptiacus*). As outgroups, we also included 12 other laurasiatherian mammals: *Ailuropoda melanoleuca* (giant panda), *Bos taurus* (cow), *Canis familiaris* (dog), *Equus caballus* (dog), *Erinaceus europaeus* (European hedgehog), *Felis catus* (cat), *Mustela furo* (ferret), *Ovis aries* (sheep), *Sorex araneus* (common shrew), *Sus scrofa* (pig), *Tursiops truncatus* (bottlenose dolphin) and *Vicugna pacos* (alpaca). Although additional genome and transcriptome data were available for bats at the time of the analysis (e.g. refs 13, 42–44), we decided to focus only on species whose genome was either Sanger sequenced or sequenced using high-throughput technologies at very high depth of coverage (>75X). Our reasoning was based upon previous findings showing that gene prediction and/or annotation errors could inflate estimates of gene turnover for taxa with low coverage draft genomes^30^, a type of bias that we wanted to alleviate -or at least minimize- in downstream analyses. To this end, we also assessed gene annotation completeness of our 20 proteomic datasets using the Benchmarking Universal Single-Copy Orthologs (BUSCO) software, based on a 3,300 gene set conserved across vertebrates^18^.

If alternative protein isoforms were present for a given locus in any of the above proteomes, we only kept the first splicing variant (usually the longest). We predefined a species tree topology based on the most recent phylogenomic study of bats by Tsagkogeorga *et al*.^13^, and ran the OMA pipeline under default parameters. To control that the inference of gene gains and losses was not biased by the predefined input phylogeny, we repeated our analysis using a tree topology estimated *de novo* directly from the proteomic data using OMA (parameter “SpeciesTree” set to “estimate” in the parameter file), as well as six proposed alternative species tree topologies for the diversification of laurasiatherian orders (Supplementary Figure S1). To map events of gene contraction and expansion at specific branches of the tree, the OMA hierarchical group output was parsed with HAM, a Python program developed by the authors available at http://lab.dessimoz.org/ham, inferring the placement of gene gains, duplications and losses using a parsimony criterion (with all types of events equally weighted).

### Maximum likelihood estimation of the rate of gene turnover

To estimate the rate of HOG expansion and contraction in bats, we analysed the HOG data inferred from the OMA pipeline using the software CAFE 3^31^. For each HOG, we counted the number of genes present for each sampled genome in a given group of homologues, and converted these HOG counts at the tips of our tree into a CAFE formatted dataset. Again we used the species tree topology of Tsagkogeorga *et al*.^13^ (consistent with the *de novo* tree inferred by OMA), together with divergence times taken from Meredith *et al*.^45^ and from www.timetree.org to build the following reference species tree: (((*R*. *aegyptiacus*: 24, (*P*. *alecto*: 12, *P*. *vampyrus*: 12): 12): 42, (*M*. *natalensis*: 45, (*E*. *fuscus*: 25, (*M*. *davidii*: 14, (*M*. *lucifugus*: 9, *M*. *brandtii*: 9): 5): 11): 20): 21): 14, ((*F*. *catus*: 55, (*C*. *l*. *familiaris*: 46, (*M*. *p*. *furo*: 40, *A*. *melanoleuca*: 40): 6): 9): 24, (*E*. *caballus*: 78, (*V*. *pacos*: 65, (*S*. *scrofa*: 64, (*T*. *truncatus*: 56, (*O*. *aries*: 26, *B*. *taurus*: 26): 30): 8): 1): 13): 1): 1).

To accommodate errors potentially present in our HOGs dataset, we used the caferror.py script of the CAFE 3 software package, which estimates the error in a dataset without *a priori* information on the error distribution. We ran CAFE with the error estimation on HOGs with at least one gene present in the most recent common ancestor of Scrotifera (the clade uniting bats, carnivores and ungulates). We also used the option –s in caferror.py in order to obtain estimates of error for each of our 18 mammalian genomes separately (excluding insectivores used as outgroups) in addition to the average global error estimate across all species of our phylogeny.

### Testing for accelerated evolution in bats

To assess the rate of gene turnover in bats as well as in other closely related mammals, we fitted to our data three birth-and-death models of gene family size evolution^46^: (i) a null model in which we defined a single global evolutionary rate of gene gain and loss (λ) across our tree; (ii) a two-lambda model, in which we specified a different rate of gene family turnover for the bat clade (λ_bats_) compared to the rest of the mammalian phylogeny (λ_0_); and (iii) a three-lambda model, in which we specified a different rate of gene family turnover for non-echolocating Old World fruit bats (suborder Yinpterochiroptera, λ_owf_) and echolocating forms (suborder Yangochiroptera, λ_yan_), and a third rate for the rest of the tree (λ_0_). We ran all models five times to confirm convergence to a single global maximum, allowing for separate parameterization of the rate of gene birth (λ) and gene death (μ, where λ ≠ μ) or assuming one single parameter for the average turnover of genes within a HOG (λ = μ). Analyses were repeated after correction for global error in the data, and after accounting for errors in each species separately. The p-value threshold in all runs was again specified at 0.01, and analyses were restricted to families with at least one gene in the root of the Scrotifera clade in the reference tree. To account for multiple testing, we followed the Benjamini & Hochberg’s procedure^47^ that corrects the false discovery rate (FDR). To compare estimated rates of gene change in bats to other mammalian lineages, analyses were repeated with the Carnivora and Cetartiodactyla as focal groups, respectively (data not shown).

To assess the significance of the observed HOG size differences among bats and other laurasiatherian genomes, we generated 5,000 simulated datasets under the global λ estimate after correcting for each individual species’ error (using the command *genfamily* in CAFE). This dataset was subsequently used to build a null distribution of likelihood ratios under the model of a global lambda versus one with two-lambda values assuming two independent rates, one for bats and one for the other mammals. Differences in the model fit were considered significant if they fell outside of the 95% of the null distribution.

### Functional annotation

To annotate HOGs in terms of proteins and their putative role in species biology, we first interrogated protein information available in UniProtKB database^26^. We selected one representative protein for each HOG, preferably from the cow when present, else from dog, cat or horse, as these species appeared to have better annotation records compared to the rest of our sampled mammals. Then, for a given list of HOGs of interest, we used the collected protein identifiers as queries in UniProtKB to infer functional groups based on GO terms, as well as based on keywords from protein annotations.

Second, we tested for enrichment in GO terms of HOGs showing gene gains and losses in the respective MRCAs of all bats, Old World fruit bats, echolocating bats, as well as in the MRCAs of Ferungulata, carnivores, and even-toed ungulates and cetaceans (Cetartiodactyla). Again, we sampled one cow sequence from each HOG, such as each HOG was linked to a cow gene and its associated GO terms as a proxy for the functional role of its members. In addition, based on the same set of identifiers we used Ensembl Biomart^48^ to retrieve human gene homologues, which are better annotated than other mammalian genes and thus could potentially offer better insights into the functional profile of our gene sets.

The most current associations of cow and human genes with GO terms were download from the Gene Ontology website (October 2016), and GO enrichment analyses were performed based on Fisher’s exact test, using the Python package GOATOOLS (https://github.com/tanghaibao/Goatools). The background population of HOGs for the GO analyses was defined separately in each test, and included all HOGs present in the parental node of the branch tested for enrichment. Finally, resulted p-values were adjusted for multiple testing using Bonferroni, Sidak, and Holm corrections, also implemented in GOATOOLS^49^.

### Correlation analysis between λ and genome size

The genome size (C-value) for each of our sampled species was obtained from the Animal Genome Size Database^7^. If the exact species did not have an entry in the database, we used an expected C-value calculated from the average C-value from other species of the same genus. Nonparametric Spearman correlation tests between genome size values, and estimates of average gene expansion, gene gain and gene loss were performed in R. To account for the phylogenetic history of the compared species, we used the comparative method phylogenetic independent contrasts (PIC) implemented in the PHYLIP package^50^.

## Electronic supplementary material

Supplementary Information

Supplementary Spreadsheets
