# Supplementary material for: Comparative genomics reveals contraction in olfactory receptor genes in bats
Source: Sci Rep. 2017 Mar 21;7:259. doi: 10.1038/s41598-017-00132-9 (PMC5427940; doi:10.1038/s41598-017-00132-9)
Supplement: Supplementary file 1 — Supplementary Information [file 41598_2017_132_MOESM1_ESM.pdf]

Supplementary Information

Comparative genomics reveals contraction in olfactory receptor genes in bats

Georgia Tsagkogeorga[1,*], Steven Müller[2], Christophe Dessimoz[2,3,4] and Stephen J Rossiter[1]

[1]School of Biological & Chemical Sciences, Queen Mary University of London, Mile End Road, London E1 4NS, United Kingdom

[2]University College London, Gower Street, London WC1E 6BT, UK

[3]University of Lausanne, Biophore, 1015 Lausanne, CH

[4]Swiss Institute of Bioinformatics, Biophore, 1015 Lausanne, CH

[*]Corresponding author

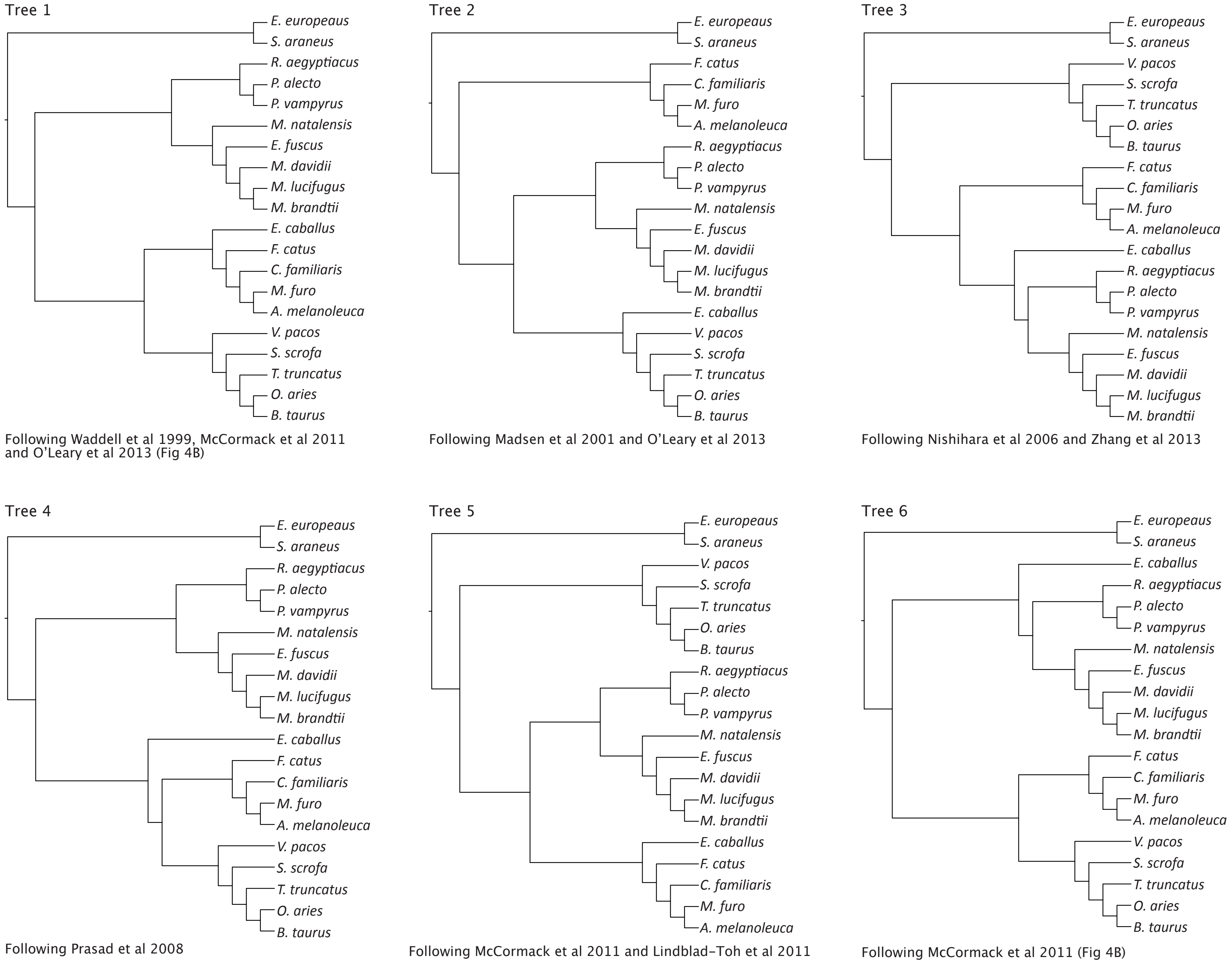

Figure S1. Alternative laurasiatherian phylogenies used in OMA analyses.

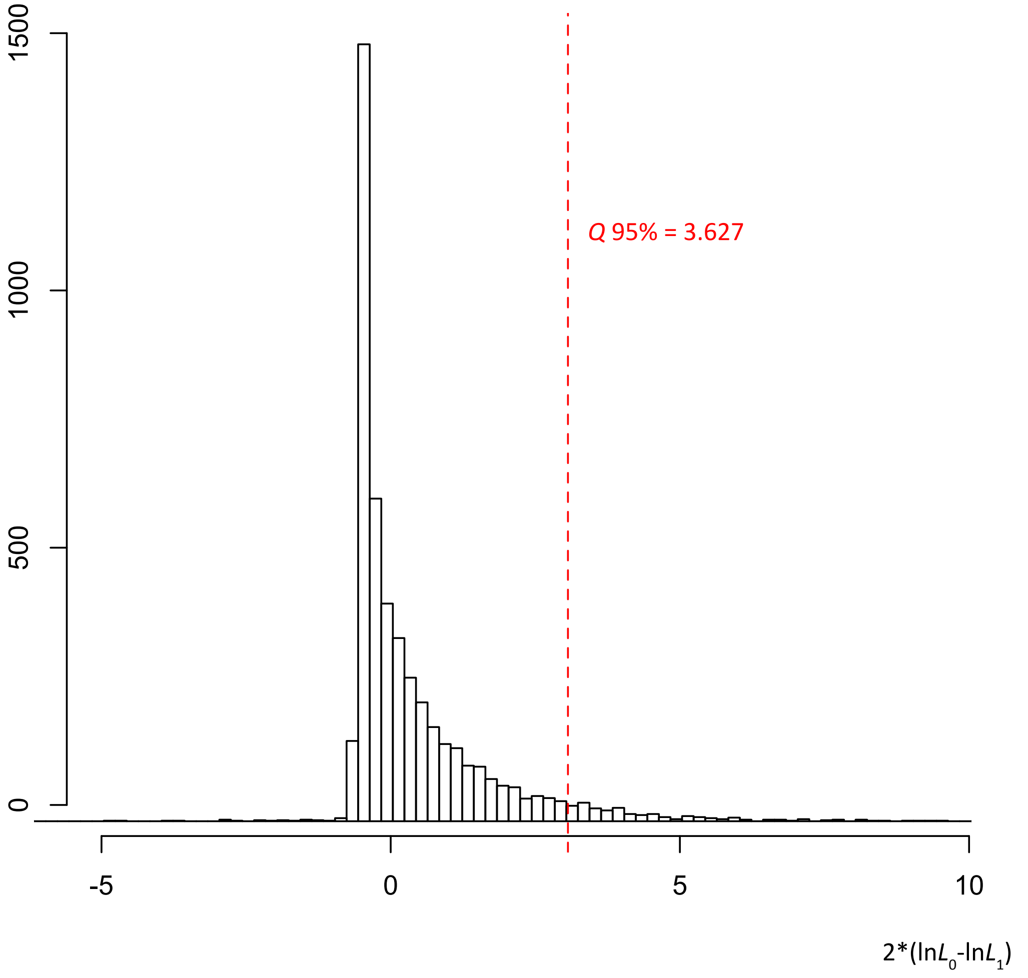

Figure S2. Null distribution of likelihood ratios based on 5,000 simulated datasets under the model of a global lambda versus one with two-lambda values assuming two independent rates, one for bats and one for the other mammals.

Table S1. Assessment of laurasiatherian proteome completeness using Benchmarking Universal Single-Copy Orthologs (BUSCO).

**Species proteome**	**Complete**	**Duplicated**	**Fragmented**	**Missing**
Pteropus alecto	98%	0.80%	1.00%	0.60%
Rousettus aegyptiacus	98%	1.60%	1.00%	0.50%
Mustela putorius furo	97%	1.50%	1.70%	1.00%
Eptesicus fuscus	97%	1.60%	1.70%	0.50%
Bos taurus	97%	1.90%	1.60%	0.50%
Ailuropoda melanoleuca	97%	2.10%	1.80%	0.30%
Canis familiaris	97%	3.80%	1.80%	0.40%
Miniopterus natalensis	96%	1.00%	2.90%	0.60%
Felis catus	96%	1.20%	2.80%	0.50%
Ovis aries	96%	2.40%	2.10%	0.90%
Equus caballus	95%	1.00%	3.80%	0.40%
Tursiops truncatus	94%	0.80%	4.20%	1.40%
Pteropus vampyrus	93%	0.70%	5.10%	1.50%
Myotis brandtii	93%	3.30%	4.90%	1.30%
Myotis lucifugus	93%	4.10%	3.10%	3.00%
Myotis davidii	92%	1.50%	4.90%	2.00%
Sus scrofa	83%	6.10%	6.80%	10.00%
Erinaceus europaeus	62%	0.70%	17.00%	19.00%
Vicugna pacos	57%	0.20%	12.00%	29.00%
Sorex araneus	55%	0.50%	14.00%	29.00%

Table S2: HOG size changes along each branch of the laurasiatherian phylogeny.

Parent Node	Daughter Node	# Ancestral genes	# Ancestral genes remaining identical	# Ancestral genes lost	# Ancestral genes expanded	# Multicopy genes
**Scrotifera**	**Chiroptera**	**20,846**	**18,602**	**1,053**	**1,191**	**2,678**
**Chiroptera**	**Old World fruit bats**	**21,411**	**18,573**	**2,272**	**566**	**1,283**
Old World fruit bats	*P. alecto* + *P. vampyrus*	19,883	18,667	994	222	461
**Chiroptera**	**Yangochiroptera**	**21,411**	**19,469**	**1,467**	**475**	**1,076**
Yangochiroptera	*E. fuscus* + Myotinae	20,632	19,766	501	365	796
E. fuscus + Myotinae	Myotinae	20,635	19,526	753	356	790
Myotinae	*M. lucifugus* + *M. brandtii*	20,444	19,216	785	443	1,026
**Scrotifera**	**Ferungulata**	**20,846**	**19,174**	**409**	**1,263**	**2,759**
Ferungulata	Carnivora	22,544	19,160	2,638	746	1,671
Carnivora	*A. melanoleuca* + *C. familiaris* + *M. furo*	20,933	19,877	807	249	536
A. melanoleuca + *C. familiaris* + *M. furo*	*A. melanoleuca* + *M. furo*	20,460	19,241	1,024	195	435
Ferungulata	Euungulata	22,544	21,009	925	610	1,331
Euungulata	Cetartiodactyla	22,431	21,569	591	271	562
B. taurus + *S. scrofa* + *O. aries* + *T. truncatus*	*B. taurus* + *O. aries* + *T. truncatus*	21,973	20,374	1,405	194	408
B. taurus + *O. aries* + *T. truncatus*	*B. taurus* + *O. aries*	20,786	19,233	1,131	422	1,095
Cetartiodactyla	*B. taurus* + *S. scrofa* + *O. aries* + *T. truncatus*	22,177	20,844	859	474	1,046

Table S3: HOG functional annotation overview.

Parent	Scrotifera	Chiroptera	Chiroptera	Scrotifera	Ferungulata	Euungulata
**Daughter Node**	**Chiroptera**	**Old World fruit bats**	**Yangochiroptera**	**Ferungulata**	**Carnivora**	**Cetartiodactyla**
**# Ancestral genes**	20,846	21,411	21,411	20,846	22,544	22,431
**# Genes remaining identical**	18,602	18,573	19,469	19,174	19,160	21,569
**# Multicopy genes from expansions**	2,678	1,283	1,076	2,759	1,671	562
**# HOGs showing expansion**	**1,191**	**566**	**475**	**1,263**	**746**	**271**
**GO annotated based on members using UniProtKB**						
**#HOGs**	830	377	340	922	561	185
**% HOGs**	**69.7%**	**66.6%**	**71.6%**	**73.0%**	**75.2%**	**68.3%**
**GO annotated based on human homologs using GOATOOLS**						
**# HOGs**	1,040	486	420	1,074	610	233
**% HOGs**	**87.3%**	**85.9%**	**88.4%**	**85.0%**	**81.8%**	**86.0%**
**# Ancestral genes lost**	**1,053**	**2,272**	**1,467**	**409**	**2,638**	**591**
**# HOGs showing contraction**	1,004	2,041	1,419	373	2,501	546
**GO annotated based on members using UniProtKB**						
**#HOGs**	656	1,057	630	195	1,198	339
**% HOGs**	**65.3%**	**51.8%**	**44.4%**	**52.3%**	**47.9%**	**62.1%**
**GO annotated based on human homologs using GOATOOLS**						
**# HOGs**	698	1,205	690	238	1,401	382
**% HOGs**	**69.5%**	**59.0%**	**48.6%**	**63.8%**	**56.0%**	**70.0%**

Table S4: Functional annotation of HOGs showing gene expansion and contraction in the MRCA of bats based on GO terms in UniProtKB.

**GO term**	**Expansions Annotated 830/1,191**	**Contractions Annotated 656/1,004**	**All member loss 119/201**
Cellular process	629	546	0
Single-organism process	535	455	97
Biological regulation	500	403	92
Regulation of biological process	476	383	92
Metabolic process	366	312	27
Response to stimulus	294	289	83
Multicellular organismal process	271	229	77
Single organism signaling	211	214	76
Developmental process	191	122	7
Cellular component organisation or biogenesis	182	157	11
Localization	179	144	8
Immune system process	80	51	4
Multi-organism process	59	29	3
Locomotion	44	22	2
Reproduction	43	20	2
Reproductive process	43	20	2
Cell adhesion	41	30	1
Behavior	21	10	1
Growth	14	16	2
Rhythmic process	10	2	0
Presynaptic process involved in chemical synaptic transmission	4	2	0
Detoxification	3	2	0
Cell killing	2	0	0
Cellular oxidant detoxification	0	2	0
Cartilage condensation	0	1	0
Antibody-dependent cellular cytotoxicity	0	1	0
Developmental growth	0	0	2
Locomotory behavior	0	0	1

Table S5: Functional annotation of HOGs showing gene expansion and contraction in the MRCA of bats based on keyword terms in UniProtKB.

**Keyword**	**Expansions Annotated 192/1,191**	**Contractions Annotated 206/1,004**	**Lost in MRCA Bats 73/201**
Olfaction	0	92	68
Transport	51	36	1
Transcription	29	23	0
mRNA splicing	8	12	0
Ubl conjugation pathway	6	7	1
Differentiation	2	7	1
Lipid metabolism	4	6	0
Cell cycle	6	5	0
Cell adhesion	11	4	0
DNA repair	6	4	0
Apoptosis	4	3	0
DNA replication	4	2	0
Autophagy	2	2	0
Glycolysis	1	2	0
Myogenesis	0	2	1
Neurogenesis	2	1	0
Heme biosynthesis	2	1	0
DNA recombination	1	1	0
Purine biosynthesis	1	1	0
Wnt signaling pathway	1	1	0
Peroxisome biogenesis	1	1	0
Sensory transduction	43	0	0
Immunity	5	0	0
Protein biosynthesis	5	0	0
Chemotaxis	3	0	0
Antiviral defense	2	0	0
Exocytosis	2	0	0
Carbohydrate metabolism	2	0	0

Iron storage	1	0	0
Mast cell degranulation	1	0	0
GPI-anchor biosynthesis	1	0	0
Neurotransmitter biosynthesis	1	0	0
Porphyrin biosynthesis	1	0	0
DNA synthesis	1	0	0
Inflammatory response	1	0	0
Digestion	1	0	0
Branched-chain amino acid catabolism	1	0	0
tRNA processing	1	0	0
Ubiquinone biosynthesis	1	0	0
Biological rhythms	1	0	0
Cilium biogenesis/degradation	1	0	0
Transcription regulation	0	0	3

Table S6-S13: See separate .xls spreadsheet

Table S14: Functional annotation of HOGs showing gene expansion and contraction in the MRCA of Old World fruit bats and Yangochiroptera based on GO terms in UniProtKB.

	**Expanded**		**Contracted**	
**GO term**	**MRCA Old World fruit bats Annotated 377/566**	**MRCA Yangochiroptera Annotated 340/475**	**MRCA Old World fruit bats Annotated 1,057/1,263**	**MRCA Yangochiroptera Annotated 630/746**
Cellular process	280	256	762	465
Biological regulation	261	213	622	401
Single-organism process	254	206	659	404
Regulation of biological process	250	201	590	390
Response to stimulus	173	127	362	251
Metabolic process	137	179	458	211
Multicellular organismal process	135	105	313	217
Single organism signaling	127	75	241	198
Developmental process	80	81	212	96
Localization	76	68	207	100
Cellular component organisation or biogenesis	73	79	237	90
Immune system process	32	29	100	44
Multi-organism process	24	23	76	41
Locomotion	23	20	39	20
Reproductive process	20	19	47	25
Reproduction	20	19	47	25
Cell adhesion	18	17	0	0
Behavior	9	9	22	13
Growth	8	7	20	5
Rhythmic process	4	4	8	7
Presynaptic process involved in chemical synaptic transmission	3	1	6	2
Cartilage condensation	1	0	2	0
T cell mediated cytotoxicity	1	0	0	0

Biological adhesion	0	0	46	18
Cell killing	0	0	2	0
Cellular oxidant detoxification	0	0	2	0
Iodide peroxidase activity	0	1	0	0
Detoxification	0	0	2	5
Leukocyte mediated cytotoxicity	0	0	0	1
Menstruation	0	0	0	1

Tables S15-S21: See separate .xls spreadsheet

Table S22. Birth-and-death (BD) model fit on 18,698 HOGs present in the MRCA of Scrotifera (Bats + Carnivores + Perissodactyla + Cetartiodactyla).

BD Model	λ_0	λ_1	μ_0	μ_1	-lnL	AIC
**No error correction**						
1-lambda	0.0017	-	-	-	184,044.80	368,091.60
2-lambda, 1=Bats	0.0017	0.0018	-	-	184,028.60	368,061.20
2-lambda, 1=Carnivora	0.0019	0.0011	-	-	182,933.79	365,871.57
2-lambda, 1=Cetartiodactyla	0.0015	0.0023	-	-	182,934.14	365,872.27
1-lambdamu	0.0004	-	0.0030	-	167,963.90	335,931.81
2-lambdamu, 1=Bats	0.0003	0.0004	0.0029	0.0031	167,925.19	335,858.38
2-lambdamu, 1=Carnivora	0.0004	0.0002	0.0034	0.0019	166,880.44	333,768.87
2-lambdamu, 1=Cetartiodactyla	0.0003	0.0004	0.0025	0.0040	166,891.10	333,790.21
**Global error correction, ε=0.0635**						
1-lambda	0.0017	-	-	-	184,044.80	368,091.60
2-lambda, 1=Bats	0.0017	0.0018	-	-	184,028.60	368,061.20
2-lambda, 1=Carnivora	0.0019	0.0011	-	-	182,933.79	365,871.57
2-lambda, 1=Cetartiodactyla	0.0015	0.0023	-	-	182,934.14	365,872.27
1-lambdamu	0.0004	-	0.0030	-	167,963.90	335,931.81
2-lambdamu, 1=Bats	0.0003	0.0004	0.0029	0.0031	167,925.19	335,858.38
2-lambdamu, 1=Carnivora	0.0004	0.0002	0.0034	0.0019	166,880.44	333,768.87
2-lambdamu, 1=Cetartiodactyla	0.0003	0.0004	0.0025	0.0040	166,891.10	333,790.21
**Individual species error correction, ε=[0, 0.4126]**						
1-lambda	0.0008	-	-	-	162,987.48	326,008.97
2-lambda, 1=Bats	0.0008	0.0008	-	-	162,987.36	326,010.73
2-lambda, 1=Carnivora	0.0008	0.0007	-	-	162,954.29	325,944.58
2-lambda, 1=Cetartiodactyla	0.0008	0.0007	-	-	162,954.29	325,944.58
1-lambdamu	0.0001	-	0.0016	-	155,133.69	310,303.38
2-lambdamu, 1=Bats	0.0001	0.0001	0.0017	0.0015	155,107.15	310,254.29
2-lambdamu, 1=Carnivora	0.0001	0.0001	0.0017	0.0014	155,033.81	310,107.63
2-lambdamu, 1=Cetartiodactyla	0.0001	0.0001	0.0016	0.0019	155,027.65	310,095.30
